# An unexpected role for the brain in the pathogenesis of diabetic ketoacidosis

**DOI:** 10.1172/JCI196357

**Published:** 2025-08-01

**Authors:** Zaman Mirzadeh, Gregory J. Morton, Irl B. Hirsch, Michael W. Schwartz

**Affiliations:** 1 Department of Neurosurgery, Barrow Neurological Institute, Phoenix, Arizona, USA.; 2 University of Washington Medicine Diabetes Institute, Department of Medicine, Seattle, Washington, USA.

In 2011, we and others made an unexpected discovery: In rodent models of uncontrolled type 1 diabetes (T1D), the most severe manifestations of insulin deficiency — hyperglycemia and diabetic ketoacidosis (DKA) — can be fully reversed by infusing the adipocyte hormone leptin directly into the brain (1, 2). Although we were excited by this unprecedented finding, it was incompatible with the prevailing view that DKA is purely a consequence of insulin deficiency in the periphery. There was simply no context within which we could understand how an adipocyte hormone could act in the brain to accomplish such a feat.

Here, we provide a modern framework for understanding how the brain, under the influence of leptin (and without the need for insulin), can reverse the most severe manifestations of uncontrolled T1D. Viewed through the lens of this new framework, our unexpected finding now seems totally predictable. Key to this understanding is the brain’s role in ensuring that the body’s fuel needs are met during periods of fasting.

## Meeting the body’s fuel needs

After ingested nutrients have been absorbed, the body’s energy needs must be met by mobilizing fuels in the necessary amounts. The brain plays an important role in this process: it has the unique ability to detect when body fuel stores are threatened and, in response, mobilize fuels for use by cells throughout the body (3–5). The predominant fuels are glucose and ketones, synthesized primarily by the liver, and free fatty acids (FFAs) and glycerol, mobilized by hydrolysis of adipose tissue triglycerides (lipolysis). Virtually all cells in the body can meet their energy requirements by metabolizing one or more of these substrates.

**Figure 1:**
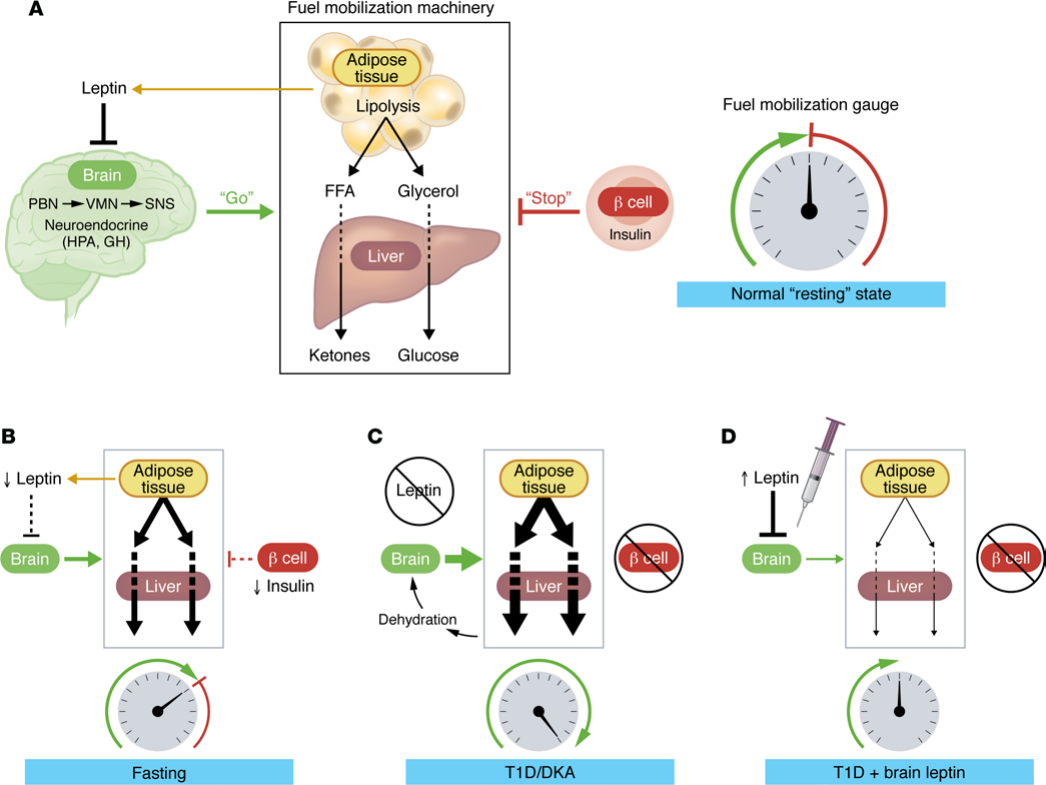
Opposing roles of brain and pancreas in the control of fuel mobilization. (**A**) Under fed conditions, fuel mobilization is inhibited both by the action of leptin in the brain and by the inhibitory effect of insulin (secreted by pancreatic β cells) on liver and adipose tissue. HPA, hypothalamic-pituitary-adrenal; GH, growth hormone. (**B**–**D**) In the fasted state (**B**), low leptin levels trigger multiple neuroendocrine and autonomic mechanisms (secretion of glucagon, increased sympathetic outflow) to promote mobilization of fuels — primarily, glucose and ketones from the liver and FFAs and glycerol from adipose tissue. These responses are constrained by insulin. In T1D (**C**), severe insulin deficiency causes leptin deficiency and eliminates the normal brake on fuel mobilization. Brain perception of fuel depletion therefore triggers fuel mobilization that is unopposed by either the central action of leptin or the peripheral action of insulin, leading to progressive hyperglycemia and DKA. By blocking the perception of fuel deficiency, leptin delivery directly into the brain (**D**) restores circulating fuel levels to normal, despite ongoing, severe insulin deficiency.

To promote fuel mobilization, the brain orchestrates both neuroendocrine (e.g., enhanced secretion of growth hormone, cortisol, glucagon, and epinephrine) (6–8) and autonomic (increased sympathetic nervous system [SNS] outflow to liver, adipose tissue, and pancreas) (9–11) responses to stimulate gluconeogenesis, ketogenesis, and lipolysis. This multi-organ symphony is biochemically interconnected in the liver, where glycerol and FFAs derived from adipose tissue lipolysis are used as substrates for gluconeogenesis and ketogenesis, respectively (Figure 1).

While the brain drives these responses, the level of circulating insulin must also remain low, because insulin inhibits gluconeogenesis, ketogenesis, and lipolysis (12), thus providing a physiological brake on fuel mobilization. In normal animals, the nutrient-responsive nature of insulin secretion constrains fuel mobilization, such that during fasting, the rate at which fuels enter the circulation matches the rate at which they are cleared from the circulation. The net result is that blood levels of glucose, ketones, FFAs, and glycerol are stably maintained within normal limits. As detailed below, however, the inability to secrete insulin, due for example to β cell injury, in combination with brain-driven fuel mobilization, creates a vicious cycle fundamental to the pathogenesis of uncontrolled hyperglycemia and DKA.

## Communicating fuel store status to the brain

While information regarding the sufficiency of body fuel stores is conveyed to the brain in various ways, the level of circulating leptin plays an outsized role (5). During fasting, lower insulin levels promote lipolysis in adipocytes, which in turn rapidly halts leptin secretion (13). The rapid decline in circulating levels of both hormones conveys to the brain that body fuel stores are under threat. In response, the brain engages its fuel-mobilizing machinery. Importantly, the brain interprets a low leptin signal as evidence of depleted fuel stores regardless of the actual status of stored fuel. Indeed, individuals with genetic leptin deficiency experience an unrelenting drive to both consume and mobilize fuels (14, 15), which can result in obesity and a form of type 2 diabetes (16, 17).

## Brain responses to fuel depletion

In addition to the neuroendocrine (6–8, 18) and autonomic mechanisms (9–11) noted above, brain-driven responses to the perception of fuel depletion include suppression of the reproductive (to avert the energy costs of reproduction) (19) and thyroid (to reduce thermogenesis) axes (20). Also, neurocircuits that drive virtually all aspects of feeding behavior are activated (increased food seeking and consumption; enhanced rewarding and reduced satiating properties of food) (21). As might be expected, leptin deficiency is a key driver of each of these responses to fuel depletion, as revealed by leptin replacement studies in fasted animals and humans (22, 23).

## Impact of uncontrolled T1D on fuel mobilization

In T1D, autoimmune destruction of pancreatic β cells causes severe insulin deficiency, setting in motion a series of catastrophic consequences. While insulin deficiency directly reduces tissue glucose uptake, of greater concern is that the normal brake on fuel mobilization provided by insulin no longer exists. As insulin deficiency begets leptin deficiency, leptin levels also drop precipitously — a powerful signal to the brain that fuel stores are depleted, regardless of how much fuel is actually present. An irresistible stimulus to eat, leptin deficiency is also implicated in the genesis of “diabetic hyperphagia” (24). In animal models of uncontrolled T1D, daily food intake typically increases two-fold.

The net result is that fuel mobilization by the brain is robustly and relentlessly activated (by leptin deficiency), and because insulin cannot be secreted, there are no constraints on the mobilization process. The situation can be conceptualized as stepping on the fuel mobilization accelerator pedal without the safeguard provided by the insulin brake pedal, all of which is made worse by excessive caloric intake (Figure 1).

## Progression to DKA

As the circulation becomes flooded with glucose, ketones, FFAs, and glycerol, volume contraction owing to osmotic diuresis begins to limit renal perfusion and hence renal glucose clearance, exacerbating hyperglycemia. As ketones are secreted from the liver in the form of ketoacids, the dehydrating effect of hyperglycemia is accompanied by growing metabolic acidosis, a progression that poses a serious threat to survival. Responding to this threat, the brain activates the sympathoadrenal system, releasing epinephrine into the circulation, which only further exacerbates gluconeogenesis, ketogenesis, and lipolysis. The result is a vicious cycle wherein the stress of dehydration, volume contraction, and acidosis exacerbate the underlying fuel mobilization crisis, exacerbating the dehydration and acidosis.

## Targeting the brain to break the DKA vicious cycle

Over a century ago, the discovery of insulin was heralded as a miracle because it could halt the unrestrained metabolic derangements that drive DKA. The key point of this article, however, is that based on the above considerations, we can consider the hypothesis that this pathogenic cascade is driven as much by the brain’s perception of fuel deficiency as it is by insulin deficiency. One appealing aspect of this viewpoint is that it lends itself to critical hypothesis testing. For example, it follows logically that if the brain perceives that fuel stores *are not* depleted, the manifestations of severe insulin deficiency will be attenuated or even reversed altogether. How might we test this hypothesis?

Perhaps the best way to convince the brain that fuel stores are not depleted is to administer leptin directly into the brain. The prediction is that in response to a robust leptin signal, the brain will conclude that there is no need to mobilize fuel, and plasma levels of glucose, ketone, FFAs, and glycerol will all return into the normal range — despite ongoing, severe insulin deficiency (Figure 1). This is exactly the outcome that we observed 14 years ago (1), but at the time we lacked the physiological context needed to understand it.

To be clear, leptin administration directly into the brain is a pharmacological, rather than a physiological, intervention. Indeed, simply restoring plasma leptin levels to normal is insufficient to normalize glycemia in a rodent model of uncontrolled diabetes (although it does ameliorate ketosis) (25). Thus, we infer that (i) a pharmacological action of leptin is required to fully block the fuel mobilization response unleashed by severe insulin deficiency; and (ii) this leptin action is mediated in the brain. Given growing interest in strategies for enhancing drug delivery across the blood-brain barrier (26), noninvasive delivery of leptin into the brain may soon become feasible for human use.

## Neurocircuits governing fuel mobilization

In DKA, as in fasting, ketogenesis is dependent on the hepatic action of glucagon. In leptin-deficient states, increased SNS outflow to pancreatic islets drives glucagon secretion via a mechanism involving the parabrachial nucleus (PBN) of the brain stem (27). The PBN is part of an ascending neurocircuit that extends from the hindbrain to hypothalamic areas crucial to metabolic homeostasis, including the ventromedial nucleus (VMN) (4, 28).

In this neurocircuit, neurons projecting from the PBN to the VMN are implicated in leptin deficiency’s effect of stimulating glucagon release and hence ketogenesis in the liver (26). This assertion is based on the finding that in rodents with uncontrolled insulin-deficient diabetes, leptin action localized to the PBN is sufficient to normalize both glucagon levels and ketogenesis, presumably via projections to the VMN, despite having little effect on hyperglycemia (29). That leptin ameliorates hyperglycemia and ketogenesis via actions that are separable from one another, and involve distinct brain areas, highlights the distributed nature of the brain’s fuel-mobilizing neurocircuitry.

The VMN is also a key target for leptin’s ability to ameliorate diabetes. Although the specific neuronal cell types are still being identified, leptin receptors are concentrated in this brain area, and microinjection of even a tiny dose of leptin directly into the VMN is sufficient to reverse most manifestations of uncontrolled insulin-deficient diabetes (30). Moreover, experimental silencing of specific subsets of VMN neurons also reverses most metabolic perturbations arising from severe insulin deficiency (31, 32). From these observations, we infer that activation of these neurons is required for the most severe, acute manifestations of uncontrolled insulin-deficient diabetes. Stated differently, when the brain perceives ongoing, severe fuel depletion (e.g., in leptin deficiency), VMN neuron activation provides the obligatory response.

## Conclusion

Together, these observations highlight how the most severe metabolic consequences of uncontrolled, insulin-deficient diabetes can be reversed either by convincing the brain that fuel stores are not depleted or by silencing neurons responsible for fuel mobilization. Accordingly, translational studies are warranted to determine whether the most severe manifestations of uncontrolled T1D in humans can be ameliorated through increased leptin signaling in the brain.

In addition to shedding new light on the brain’s fundamental role in DKA pathogenesis, the evolution of research in this area illustrates how an observation that seemingly defies explanation can, over time, give way to a new understanding of a common and severe disease process.

## Version 1 08/01/2025

Electronic publication
